# Two-Dimensional-Like Amorphous Indium Tungsten Oxide Nano-Sheet Junctionless Transistors with Low Operation Voltage

**DOI:** 10.1038/s41598-019-44131-4

**Published:** 2019-05-20

**Authors:** Po-Yi Kuo, Chien-Min Chang, I-Han Liu, Po-Tsun Liu

**Affiliations:** Department of Photonics and Institute of Electro-Optical Engineering, National Chiao Tung University, Hsinchu, 30010 Taiwan

**Keywords:** Electronic devices, Electrical and electronic engineering, Electronic devices, Electrical and electronic engineering

## Abstract

In this work, we have successfully demonstrated the junctionless (JL) transistors with two-dimensional-like (2D-like) nano-sheet (NS) material, amorphous indium tungsten oxide (a-IWO), as an active channel layer. The influences of the different gate insulator (GI) materials and the scalings of GI thickness, a-IWO channel thickness, and channel lengths on the a-IWO NS JL transistors (a-IWO NS-JLTs) have been studied for the purposes of low operation voltage (gate voltage ≤2V) and high performance. The 2D-like a-IWO NS-JLTs exhibit low operation voltage, low source/drain (S/D) contact resistance (R_C_) and other key electrical characteristics, such as high field-effect mobility (μ_FE_), near ideal subthreshold swing (S.S.), and large ON/OFF currents ratio (I_ON_/I_OFF_). The remarkable device characteristics also make the proposed 2D-like a-IWO NS-JLTs promising for system-on-panel (SoP) and vertically stacked (VS) hybrid CMOS applications.

## Introduction

As devices scaling continues, Si-based ultra-thin body (UTB) field-effect transistors (FETs) and FinFET with low leakage currents and good gate controllability allow gate/channel length reduction through Fin (body) thickness scaling^1,2^. Furthermore, the single crystalline silicon (Si)/polycrystalline silicon (poly-Si)-based nanowires (NWs) and nano-sheet (NS) junctionless (JL) devices with low operation voltage and near ideal subthreshold characteristics have been proposed and demonstrated for lower thermal budgets and easier processes^3–6^. Since the gradient of a doping concentration is absent, the issues of the sharp doping profile formation and the impurities diffusion are completely eliminated^5^. Nevertheless, FETs/FinFET with sub-5nm technology node require sub-3nm-thick body for better channel controllability^7^. Most Si-based devices with such an ultra-thin body thickness will face several challenges, including the control of channel thickness (T_ch_), high source/drain (S/D) parasitic resistance (R_SD_), and uniform S/D and channel heavy doping for JL FETs devices^6^. Therefore, as Si-based CMOS scaling approaches its limits (T_ch_ < 5 nm), it is highly urgent and desirable to investigate other ultra-thin/two-dimensional (2D) channel materials with relatively wide band gaps and high mobility. Such ultra-thin/2D channel materials can potentially suppress short channel effects (SCEs) and achieve high ON/OFF currents ratio (I_ON_/I_OFF_) owing to good gate electrostatics and low leakage currents for digital circuit applications^8^.

Recently, indium oxide (InO_x_)-based transparent amorphous oxide semiconductor thin film transistors (TAOS TFTs) with wide band gaps have been developed and applied not only for display but also for other applications, like flexible electronics, optoelectronics, and mobile electronics owing to superior uniformity, low-temperature processes, and high field-effect mobility (μ_FE_)^9^. In the last few years, the vertically stacked (VS) hybrid complementary metal-oxide-semiconductor (CMOS) consisted of low-temperature *c*-axis aligned crystalline In-Ga-Zn-O (CAAC-IGZO) FETs with 60 nm technology node and p-channel Si MOSFETs with 65 nm technology node have been successfully fabricated and demonstrated^10–12^. Back-end of line (BEOL) compatible nano-scaled CAAC-IGZO FETs with ultra-low OFF-state currents (I_OFF_) and SCEs immunity are promising candidates for low power large-scale integration (LSI) and internet of things (IoT) applications^12^. Although CAAC-IGZO FETs exhibit a μ_FE_ ~10 cm^2^/V-s, the μ_FE_ and subthreshold swing (S.S.) of CAAC-IGZO FETs are degraded by increasing channel width^10^. Consequently, further improvements in μ_FE_ and the stability of the TAOS TFTs are urgently required to broaden their range of potential applications. To achieve these targets, an amorphous indium tungsten oxide (a-IWO) semiconducting material with high mobility and stability, which is free from both Ga and Zn, was studied for an alternative choice to a-IGZO TFTs^13–15^. We have successfully fabricated and demonstrated the low thermal budget 2D-like a-IWO NS JL transistors (a-IWO NS-JLTs) in bottom metal gate (BMG) configurations with high μ_FE_ ~25.3 cm^2^/V-s, near ideal S.S. ~63 mV/dec., and improved hysteresis characteristics for the first time^16^. The thickness of conductive a-IWO NS channel can be well controlled by radio-frequency (RF) magnetron sputtering at room temperature. The a-IWO NS-JLTs with metal S/D electrodes exhibit ultra-low leakage currents owing to the wider band gap of a-IWO compared to Schottky-barrier Si devices with high leakage currents^17^. Furthermore, the R_SD_ also can be significantly reduced by using metal S/D electrodes.

In this work, we will study the influences of the different gate insulator (GI) materials and the scalings of GI thickness, a-IWO channel thickness, and channel lengths on the electrical characteristics and performances of a-IWO NS-JLTs. In addition, a low power and high performance CMOS inverter based on low temperature devices is the basic and essential component in digital circuits for the pressing applications such as wearable electronics and IoT technology. Although some hybrid CMOS inverters constructed by low temperature n-channel TAOS and p-channel poly-Si TFTs had been studied and realized, the electrical characteristics of TFTs were performed with high operation voltage, poor S.S, and large I_OFF_ in these previous studies^18,19^. A conceptual VS hybrid CMOS structure consisted of BEOL compatible n-channel a-IWO NS-JLTs and p-channel poly-Si TFTs will be proposed and characterized in this work. The matched electrical characteristics of n- and p-channel devices with low operation voltage and low I_OFF_ are exhibiting the promising candidate for future VS Hybrid CMOS applications.

## Methods

**Figure 1:**
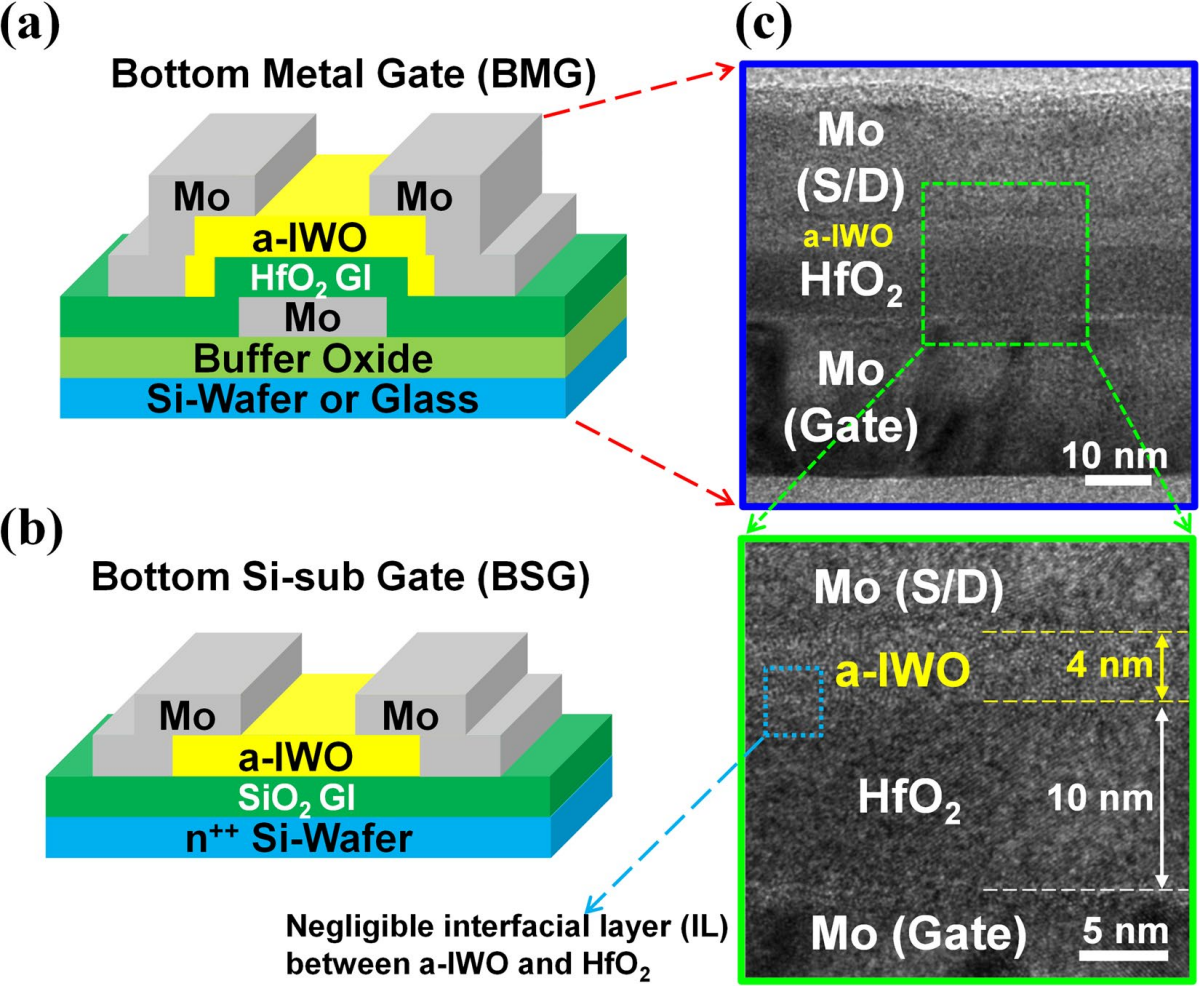
The structures diagram of (**a**) proposed BMG a-IWO NS-JLTs with HfO_2_ GI and (**b**) BSG a-IWO NS-JLTs with SiO_2_ GI; (**c**) the cross-sectional TEM images of proposed BMG a-IWO NS-JLTs.

The structure diagram of proposed BMG a-IWO NS-JLTs is schematically shown in Fig. 1(a). The proposed devices can be fabricated on Si wafers with 550-nm-thick buffer thermal SiO_2_ or on glass substrates^16^. Firstly, a layer of 25-nm-thick Mo film was deposited subsequently by the direct current (DC) sputtering and patterned as the gate electrode through photolithography. Secondly, a layer of 10-nm or 20-nm or 30-nm-thick HfO_2_ was deposited by atomic layer deposition (ALD) as the gate insulator (GI). Next, a layer of 4-nm or 10-nm-thick a-IWO channel was deposited by RF magnetron sputtering of an In-W-O (contained 4 wt.% of WO_3_) ceramic plate target at room temperature. Then, the a-IWO channel active layer was patterned through photolithography. Thirdly, a layer of 25-nm-thick Mo film was deposited by DC sputtering and patterned as the source/drain (S/D) electrodes using the lift-off technique. Finally, after channel passivation processes, contact holes to the gate and S/D electrodes were patterned and opened through photolithography.

To investigate the influences of different GI materials on the devices, the a-IWO NS-JLTs in bottom Si-sub gate (BSG) configurations with a-IWO NS channel = 4 nm were also fabricated on a heavily doped n-type Si wafer with 30-nm-thick high-quality thermal SiO_2_ GI, as shown in Fig. 1(b). Additionally, the low metal contamination Ni-induced lateral crystallization (LC-NILC) poly-Si TFTs with 50-nm-thick poly-Si channel and 10-nm-thick HfO_2_ GI were also fabricated on Si-substrates to study the OFF-state electrical characteristics for VS hybrid CMOS applications. The detail LC-NILC processes were shown in our previous work^20^.

## Results and Discussion

Figure 1(c) displays the cross-sectional transmission electron microscope (TEM) images of proposed BMG a-IWO NS-JLTs. The thickness of a-IWO NS channel and HfO_2_ GI are 4 nm and 10 nm, respectively. In general, the surface of a-IWO film deposited by means of magnetron sputtering is extremely flat^13^. The interfacial layer (IL) was consequently formed and found during deposition of HfO_2_ GI on the channel in the conventional top-gate devices. Since the deposition of HfO_2_ GI was made before the deposition of a-IWO NS channel in the proposed BMG a-IWO NS-JLTs, the IL between the HfO_2_ GI and the a-IWO NS channel is negligible, resulting in near ideal S.S. and improved hysteresis characteristics^16^.

**Figure 2:**
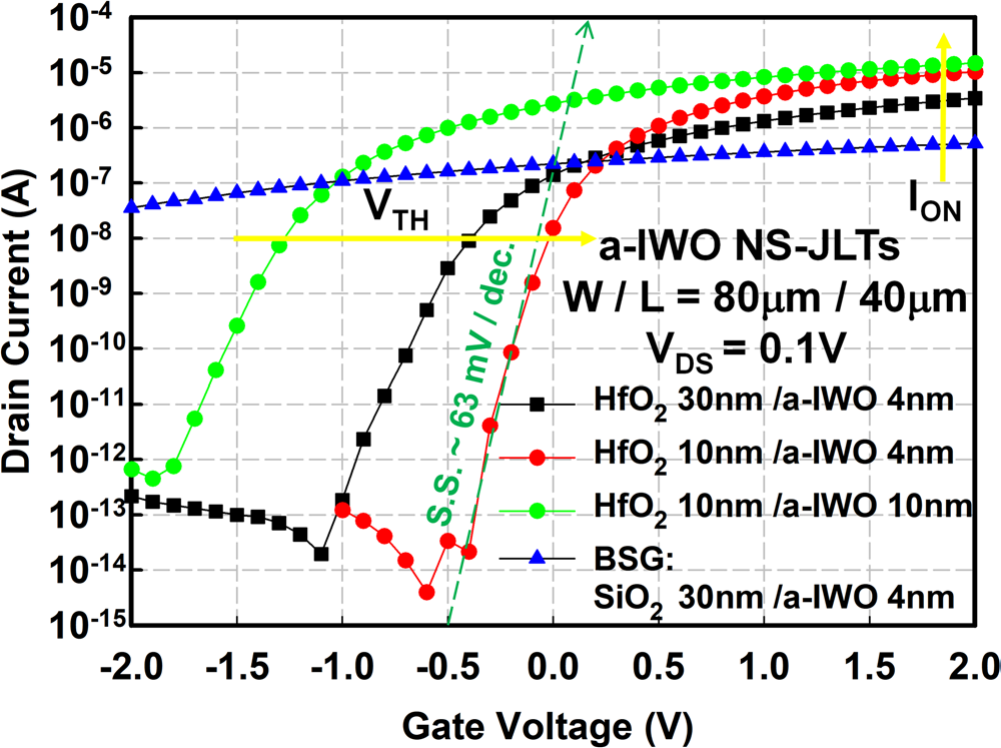
The transfer characteristics of a-IWO NS-JLTs with different GI materials, different HfO_2_ GI thickness, and different a-IWO channel thickness. The most positive V_TH_, highest I_ON_/I_OFF_, and steepest S.S. (~63 mV/dec.) are accomplished in BMG a-IWO NS-JLTs with HfO_2_ GI = 10 nm and a-IWO channel = 4 nm.

Figure 2 exhibits the transfer characteristics of a-IWO NS-JLTs with different GI materials, different HfO_2_ GI thickness, and different a-IWO channel thickness. In JL configurations, the doping or carrier concentration of the source/drain (S/D) and channel is uniform, heavy, and homogenous, which significantly reduces thermal budgets of processes and simplifies fabrication^5^. However, there are more negative V_TH_ and worse subthreshold characteristics in JL devices with thicker thickness of channel (a-IWO channel = 10 nm) or under poorer gate controls (HfO_2_ GI = 30 nm for BMG and SiO_2_ GI = 30 nm for BSG) in Fig. 2 ^21^. Among these devices, the transfer characteristics of BSG a-IWO NS-JLTs with a-IWO channel = 4 nm and SiO_2_ GI = 30 nm exhibit the weakest gate control on channel, resulting in the absence of an OFF-state within V_GS_ = −2V ~ V_GS_ = 2V. The ON-state currents (I_ON_) and S.S. of BMG a-IWO NS-JLTs are enhanced by shrinking the HfO_2_ GI thickness (HfO_2_ GI = 10 nm) thanks to the better gate controllability. Although BMG a-IWO NS-JLTs with HfO_2_ GI = 10 nm and a-IWO channel = 10 nm have the highest I_ON_ and μ_FE_, the most positive V_TH_, highest I_ON_/I_OFF_, and steepest S.S. are accomplished in BMG a-IWO NS-JLTs with HfO_2_ GI = 10 nm and a-IWO channel = 4 nm for low operation voltage applications. Therefore, we will focus the BMG a-IWO NS-JLTs with HfO_2_ GI = 10 nm and a-IWO channel = 4 nm in the latter discussions.

**Figure 3:**
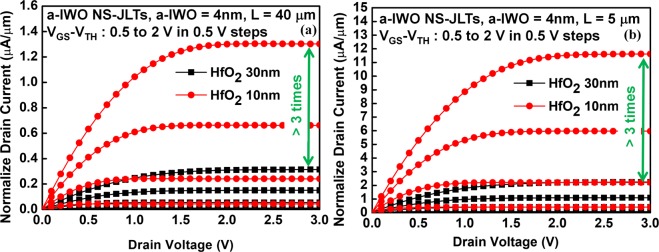
The normalized output characteristics of IWO NS-JLTs. (**a**) The a-IWO NS-JLTs with L = 40 μm and **(b**) the a-IWO NS-JLTs with L = 5 μm. The driving currents of IWO NS-JLTs with 10-nm-thick HfO_2_ are more than 3 times as large as those of IWO NS-JLTs with 30-nm-thick HfO_2_ at V_G_ − V_TH_ = 2V.

In order to enhance the I_ON_, it is necessary to improve both of μ_FE_ and the contact resistance (R_C_) between S/D metal electrodes and TAOS channel. The normalized output characteristics of a-IWO NS-JLTs with (a) channel length (L) = 40 μm and (b) L = 5 μm are shown in Fig. 3. It is noted that as the thickness of HfO_2_ GI scaling down from 30 nm to 10 nm, the driving currents of a-IWO NS-JLTs with 10-nm-thick HfO_2_ GI operated at gate overdrive voltage (V_GS_ − V_TH_) = 2V are enhanced more than 3 times of magnitude compared with the one with 30-nm-thick HfO_2_ GI. The significant enhancements in driving currents are attributable to the improvements in R_C_ between the S/D metal electrodes and the a-IWO NS channel.

**Figure 4:**
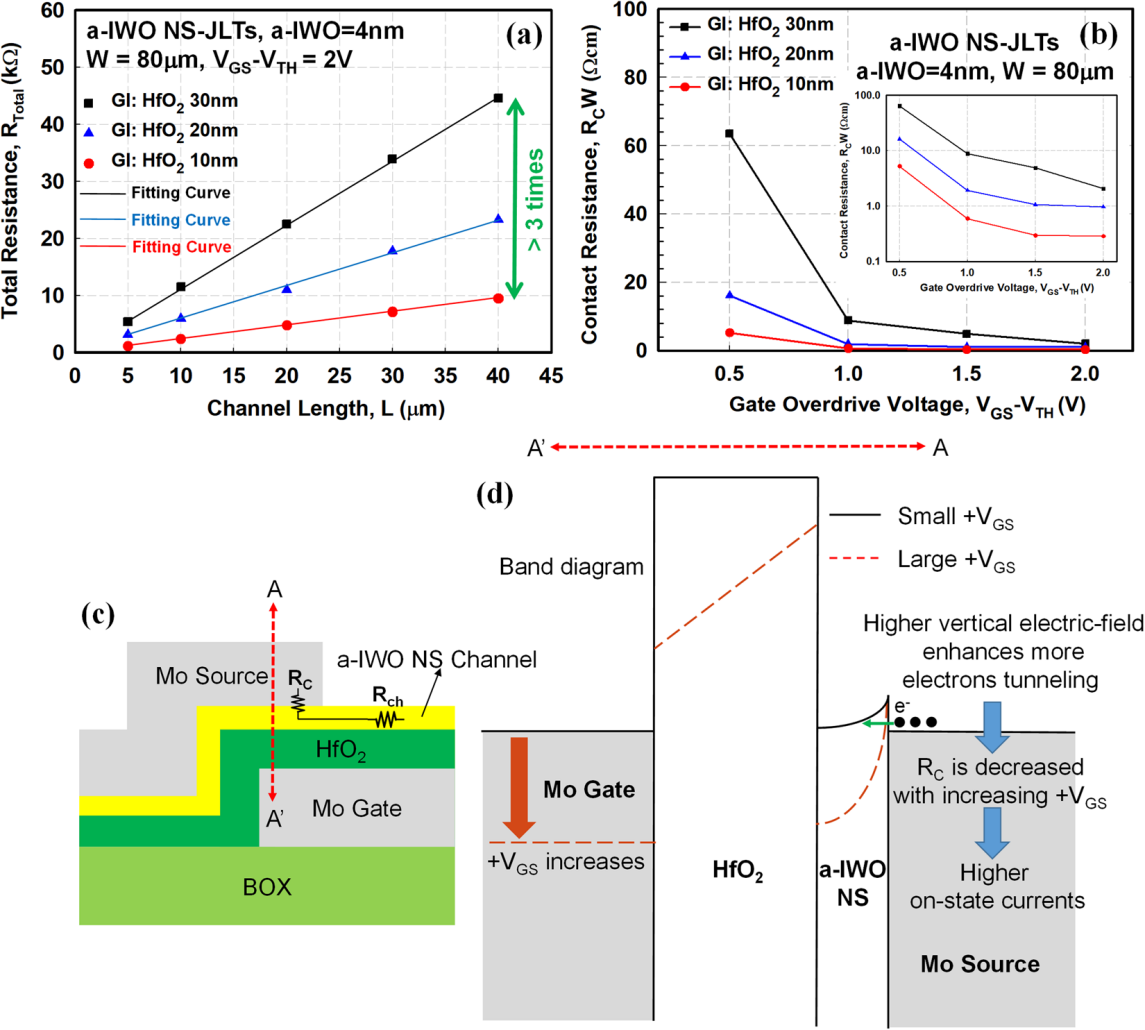
The extraction of R_C_ from the plot of R_Total_ − L and the diagrammatic explanation of R_C_ in relation to the gate voltage and the thickness of HfO_2_ GI. (**a**) The plot of R_Total_ − L at V_GS_ − V_TH_ = 2V; (**b**) the results of the extracted width-normalized R_C_ under different V_GS_ − V_TH_ in a-IWO NS-JLTs with different thicknesses of HfO_2_ GI; (**c**) the schematic structure of gate-to-source overlap region; (**d**) the illustration of band diagram along A-A’. The R_C_ is significantly improved by increasing V_GS_ − V_TH_ and scaling down the thickness of HfO_2_ GI, especially in V_GS_ − V_TH_ = 2V and HfO_2_ GI = 10 nm.

Transmission line model (TLM) measurement can be used to extract the R_C_ of metal-semiconductor junction^22^. Figure 4(a) demonstrates the plot of total resistance (R_Total_) versus channel length (L) (R_Total_ − L) at V_GS_ − V_TH_ = 2V. Figure 4(b) displays the results of the extracted width-normalized R_C_ under different V_GS_ − V_TH_ for the a-IWO NS-JLTs with different thicknesses of HfO_2_ GI. The width-normalized R_C_ in the inset of Fig. 4(b) is plotted in logarithm scale. The R_C_ is significantly improved by elevating the vertical electric-field between the gate and the overlapped S/D electrodes via increasing V_GS_ − V_TH_ and scaling down the thickness of HfO_2_ GI, especially in V_GS_ − V_TH_ = 2V and HfO_2_ GI = 10 nm.

Figure 4(c) shows the schematic structure of the gate-to-source overlap region and Fig. 4(d) illustrates of band diagram along A-A’. As shown in Fig. 4(c), when S/D electrodes contact with a-IWO NS layer, the R_C_ related to Schottky barrier between S/D metal electrode and a-IWO NS channel is formed. Since the potential energy of a-IWO NS channel at S/D electrodes contact can be modulated by the gate voltage, the Schottky barrier becomes narrower as the gate voltage increases, as shown in Fig. 4(d). Higher vertical electric-field enhances more electrons tunneling behavior in addition to thermionic electron injection^23^. The R_C_ is decreased with increasing gate voltage and scaling down the thickness of HfO_2_ GI, resulting in higher I_ON_. The electrical mechanism can be summarized that the increase of the gate voltage will decrease the Schottky barrier height and make Schottky barrier narrower, resulting in the significant reduction of the value of R_C_. If a positive gate voltage is applied, it will modify the Fermi level in a-IWO NS layer and make the a-IWO NS channel more conductive and resultantly decreasing channel resistance (R_ch_). The vertical electric-field enhanced by scaling down the thickness of HfO_2_ GI also reduces the R_C_ at the metal-semiconductor interface.

**Figure 5:**
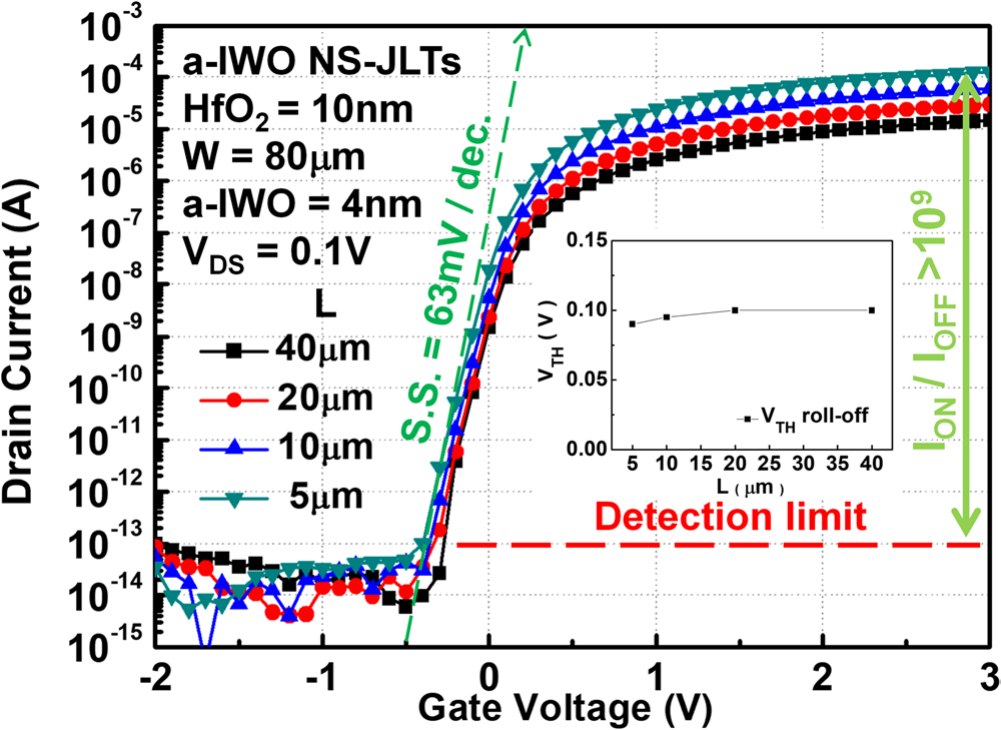
The transfer characteristics of a-IWO NS-JLTs with different channel lengths. The inset shows the extracted V_TH_ roll-off. The a-IWO NS-JLTs with very small V_TH_ roll-off exhibit high gate controllability and good SCEs immunity.

It is well known that the poly-Si transistors are BEOL compatible devices for three-dimensional integrated circuits (3-D ICs) applications^4–6^. The low-temperature BEOL TAOS TFTs and poly-Si TFTs are the suitable platforms enabling monolithic 3-D integration with hybrid CMOS technologies. One of the major challenges to integrate the TAOS and poly-Si-based CMOS technologies is on their mismatched operation voltages (V_DD_)^24^. For a low and matched operation voltage, the small S.S and real I_ON_/I_OFF_ under small gate operation voltage are critical. Figure 5 displays transfer characteristics of a-IWO NS-JLTs with different channel lengths. The extracted V_TH_ roll-off of a-IWO NS-JLTs with different channel lengths is also plotted in the inset of Fig. 5. As the channel length scaling down, the V_TH_ roll-off is a key parameter to verify the gate controllability over the channel region. The a-IWO NS-JLTs with different channel lengths have almost identical S.S. ~63 mV/dec. and similar I_OFF_ characteristics, where the I_ON_ is nearly proportional to channel length. Thus, the value of I_ON_/I_OFF_ larger than 1 × 10^9^ can be obtained for the device with 5 μm channel length at an operation conditions of V_GS_ − V_TH_ = 3V and V_DS_ = 0.1V. The a-IWO NS-JLTs with very small V_TH_ roll-off exhibit high gate controllability and good SCEs immunity thanks to the combined use of 2D-like a-IWO NS channel and thinner HfO_2_ GI in devices.

**Figure 6:**
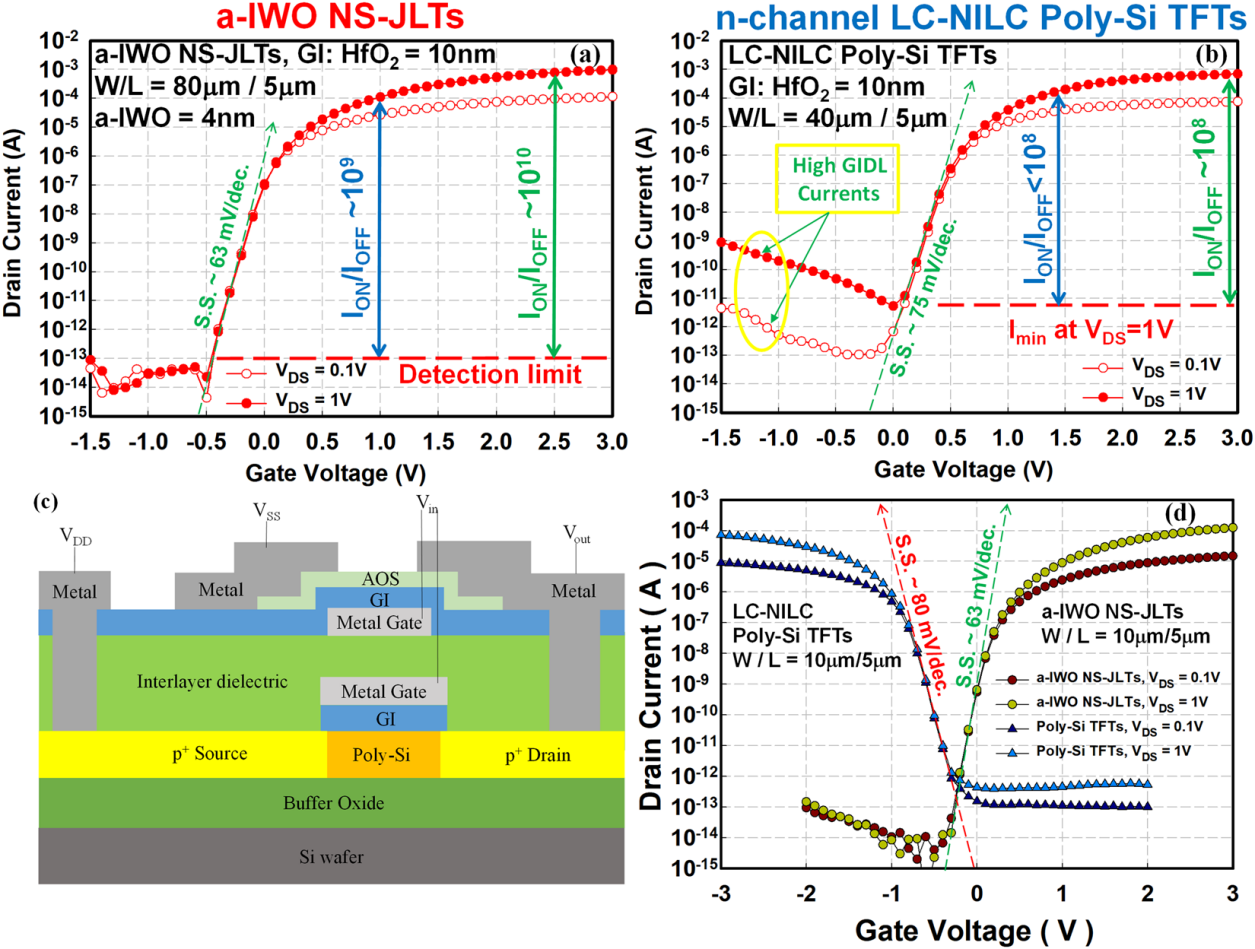
The transfer characteristics of a-IWO NS-JLTs and LC-NILC poly-Si TFTs with different V_DS_ for VS hybrid CMOS applications. (**a**) The transfer characteristics of a-IWO NS-JLTs with W/L = 80 μm/5 μm; (**b**) the transfer characteristics of n-channel LC-NILC poly-Si TFTs with W/L = 40 μm/5 μm; (**c**) the conceptual schematic of VS hybrid CMOS structure; (**d**) the transfer characteristics of a-IWO NS-JLTs and p-channel LC-NILC poly-Si TFTs. The extremely high I_ON_/I_OFF_ ~ 10^10^ at V_DS_ = 1V and V_GS_ − V_TH_ = 2.5V is accomplished in a-IWO NS-JLTs by virtue of the wide bandgap InO_x_-based NS channel. The experimental electrical characteristics of n-channel a-IWO NS-JLTs and p-channel LC-NILC poly-Si TFTs exhibit matched electrical characteristics, low operation voltage, and low I_OFF_.

For VS hybrid CMOS applications, the real I_OFF_ under large drain operation voltage is the key parameter. The transfer characteristics of a-IWO NS-JLTs with W/L = 80 μm/5 μm and n-channel LC-NILC poly-Si TFTs with W/L = 40 μm/5 μm are shown in Fig. 6(a,b), respectively. In this work, the μ_FE_ of n-channel LC-NILC poly-Si TFTs is about twice as high as that of a-IWO NS-JLTs. In order to achieve electrically-matched I_ON_, a wider channel width for the a-IWO NS-JLTs was studied. As shown in Fig. 6(a), the I_OFF_ of a-IWO NS-JLTs with W = 80 μm operated at V_DS_ = 0.1V and V_DS_ = 1V, respectively, are almost identical and smaller than the measurement detection limit (~10^−13^A). The a-IWO NS-JLTs with near ideal S.S. can be operated at low voltage. The extremely high I_ON_/I_OFF_ ~ 10^10^ at V_GS_ − V_TH_ = 2.5V and V_DS_ = 1V is accomplished in a-IWO NS-JLTs by virtue of the wide bandgap InO_x_-based NS channel. However, the n-channel LC-NILC poly-Si TFTs always suffer from higher gate-induced drain leakage (GIDL) currents and higher I_OFF_ (=I_min_ at V_DS_ = 1V) due to poly-Si film with small bandgap and grain boundaries, resulting in poorer I_ON_/I_OFF_ ~ 10^8^ at V_GS_ − V_TH_ = 2.5V and V_DS_ = 1V shown in Fig. 6(b). The a-IWO NS-JLTs with near ideal S.S., lower GIDL, and higher I_ON_/I_OFF_ are more suitable for low-power VS hybrid CMOS applications compared with the n-channel LC-NILC poly-Si TFTs.

Figure 6(c) illustrates the conceptual schematic of VS hybrid CMOS structure and Fig. 6(d) displays the transfer characteristics of a-IWO NS-JLTs and p-channel LC-NILC poly-Si TFTs. The proposed VS hybrid CMOS structure is constructed by the high-temperature p-channel LC-NILC poly-Si TFTs underneath and top low-temperature n-channel a-IWO NS-JLTs. The match of electrical characteristics with low I_OFF_ for a-IWO NS-JLTs and LC-NILC poly-Si TFTs is respectively observed to meet the requirement for hybrid CMOS applications. They are exhibiting low operation voltages and offer new opportunities of designing BEOL CMOS devices for LSI logic circuits.

The technology potential for low-temperature processes applications on a glass substrate had been demonstrated^16^. In JL devices, the path of current transport is concentrated in the center of heavy uniform doping channel, which reduces the effects at the oxide/channel interface, resulting in near ideal subthreshold characteristics^6^. It is well known that μ_FE_ is significantly decreasing as scaling channel thickness. The proposed 2D-like BMG a-IWO NS-JLTs with μ_FE_ ~ 25.3 cm^2^/V-s exhibit near ideal S.S. and improved hysteresis characteristics because of the NS channel, JL configurations, and the good interface characteristics between the HfO_2_ GI and the a-IWO NS channel^16^.

## Conclusion

In summary, we have studied the influences of the different GI materials and the scalings of GI thickness, a-IWO NS channel thickness, and channel lengths on the electrical characteristics and performances of the 2D-like a-IWO NS-JLTs. Since a-IWO NS-JLTs are fabricated by using Ga-free a-IWO thin films, the material costs can be minimized compared with typically adopted a-IGZO. The Ga- and Zn-free a-IWO NS channel layers with low cost, high mobility, and good stability could be a promising alternative to a-IGZO for the advanced oxide-based TFT technology. Also, the 2D-like BMG a-IWO NS-JLTs significantly minimize the IL thickness, resulting in near ideal S.S. and improved hysteresis characteristics. The 2D-like BMG a-IWO NS-JLTs with small V_TH_ roll-off, large I_ON_/I_OFF_, near ideal S.S., high μ_FE_, low R_C_, and low operation voltage appears highly promising potentials for system-on-panel (SoP) and VS hybrid CMOS applications in the future.
